# Use of Indocyanine Green Near-Infrared Imaging for Sentinel Lymph Node Biopsy in Early Oral Squamous Cell Carcinoma: A Pilot Study

**DOI:** 10.1007/s11307-024-01903-3

**Published:** 2024-03-05

**Authors:** Nadja A. Stenz, Gregoire B. Morand, Manuel Schoch, Jonas Werner, Gunesh P. Rajan

**Affiliations:** 1 Department of Otorhinolaryngology – Head and Neck Surgery, Luzerner Kantonsspital, Lucerne, Switzerland; 2 Department of Otorhinolaryngology, Head and Neck Surgery, University Hospital Zurich, Zurich, Switzerland; 3 Department of Otorhinolaryngology – Head and Neck Surgery, Kantonsspital St. Gallen, St. Gallen, Switzerland; 4 Otolaryngology, Head & Neck Surgery, Medical School, University of Western Australia, Perth, Australia; 5 Faculty of Health Sciences and Medicine, University of Lucerne, Lucerne, Switzerland

**Keywords:** Sentinel lymph node biopsy, Indocyanine green, Near-infrared imaging, Oral squamous cell carcinoma, ^99m^technetium

## Abstract

**Purpose:**

The current established technique for sentinel lymph node (SLN) biopsy is preoperative injection of ^99m^technetium-labeled nanosized colloids (^99m^Tc) followed by single photon emission computed tomography and standard computed tomography (SPECT/CT) with subsequent intraoperative gamma probe-guided excision of the SLN. It is however time and resource consuming, causes radiation exposure and morbidity for the patient as the injection is done in the awake patient. Recently near-infrared imaging with indocyanine green (ICG) gained importance in SLN biopsy as a faster and more convenient technique. The objective of our study was to investigate the feasibility of SLN biopsy using ICG-imaging in early oral squamous cell carcinoma (OSCC).

**Methods:**

Single-centre pilot study of five patients with early-stage OSCC. For all patients, both techniques (^99m^Tc and ICG) were performed. We injected ^99m^Tc preoperatively in the awake patient, followed by SPECT/CT imaging. Intraoperatively ICG was injected around the primary tumor. Then the neck incision was performed according to the SPECT/CT images and SLN were detected by using a gamma probe and near-infrared fluorescence imaging of the ICG-marked lymph nodes intraoperatively. The excised lymph nodes were sent to histopathological examination according to the SLN dissection protocol.

**Results:**

In all five patients sentinel lymph nodes were identified. A total of 7 SLN were identified after injection of ^99m^Tc, imaging with SPECT/CT and intraoperative use of a gamma probe. All these SLN were fluorescent and visible with the ICG technique. In two patients, we could identify additional lymph nodes using the ICG technique. Pathological analysis demonstrated occult metastasis in two of the cases.

**Conclusions:**

Our study shows that ICG-guided SLN biopsy is a feasible technique, especially in combination with conventional radioisotope method and may help for intraoperative localization of SLN. Validation studies with bigger patient cohorts are needed to prove our results.

## Introduction

Oral squamous cell carcinoma (OSCC) is an aggressive malignancy with propensity to local invasion and lymph node metastasis. 20–30% of patients with early-stage OSCC with clinically node-negative lymph node status (cN0) will have occult lymph node metastasis upon pathological examination of the lymphatic drainage [1, 2]. This information is important since regional lymph node metastasis is the most important prognostic factor of OSCC [3–5], with a decrease of about 50% in survival when present [6]. There are treatment options for early stage OSCC with a clinically N0 neck. One possibility is to perform a selective neck dissection, which may lead to overtreatment of 70–80% of the patients and morbidity [7]. Typical complications, occurring in under 15% of the cases, include marginal mandibular, vagus, hypoglossal or phrenic nerve injury, injury of the accessory nerve with shoulder pain or dysfunction, hematoma, infection, chyle leak or lymphedema, wound dehiscence and Horner’s syndrome [8]. Watch and wait and perform therapeutic neck dissection for nodal relapse is another possibility which is no longer recommended since it is detrimental to overall survival [9]. The other option is to perform a sentinel lymph node (SLN) biopsy. The concept of SLN biopsy, with identification of the first draining lymph node(s) of the regional lymph node basin was first reported in in 1977 for penile cancer and was further developed in the 1990s for OSCC [10, 11]. SLN biopsy has meanwhile proven to be a safe, effective, and reliable technique with good accuracy and comparable late recurrences and less morbidity in long-term follow-up studies in OSCC when compared to first line selective neck dissection [7, 12–15].

The current established technique for sentinel lymph node biopsy is preoperative injection of ^99m^technetium-labeled nanosized colloids (^99m^Tc) in the awake patient followed by single photon emission computed tomography and standard computed tomography (SPECT/CT) for localisation of the sentinel lymph nodes preoperatively. Subsequently intraoperative gamma probe-guided excision of the SLN is performed [16]. This technique using radioisotopes is reliable however preoperative injection around the primary tumor is painful for the patient, and the procedure consumes time and resources. As a consequence, the application is limited to cancers of the oral cavity which is accessible in the awake patient. The injection can either be done the same day of the operation requiring precise planning of imaging and surgery or alternatively in a two-step fashion (SPECT/CT some days before the surgery requiring a re-injection of radioisotope on the day of the surgery), which is logistically easier but presents the disadvantage of potential mismatch between the two injections and increasing radiation exposure for the patient and the health care personnel. Finally intraoperative localization can be challenging due to lack of visualization of the tissue and shine-through-effect, especially for floor of mouth lesions [17, 18].

Recently near-infrared imaging with indocyanine green (ICG) gained importance in SLN biopsy as a faster and more convenient technique in OSCC [19, 20]. ICG could be a good alternative to the current technique of SLN biopsy with injection of radioisotopes, as ICG accumulates in the lymph nodes and can be visualised intraoperatively [21]. ICG is a tricarbocyanine iodide with properties of a near-infrared fluorescent dye. Fluorescence of the ICG molecule is normally observed around the maximum peak of 800—850 nm. It is water soluble, has low toxicity and a low molecular weight with quick clearance by hepatic route. It can be injected and traced intraoperatively and enables an intraoperative high-resolution visualisation of the lymph nodes and lymphatic tissue [17, 21, 22]. After injection of the ICG dye in the peritumoral region it takes only a couple of minutes for the ICG to follow the lymphatic vessels and reach the first draining lymph nodes. It provides visual feedback with high area of fluorescence, indicating the precise localisation of the SLN. Especially in cervical cancer and melanoma patients it has been shown to yield good results in sentinel lymph node biopsy [22].

The objective of this study is to investigate the use of near-infrared imaging and ICG in patients with early OSCC and a clinically N0 neck and to confirm its feasibility compared with the conventional radioisotope guided SLN biopsy method.

## Material and Methods

### Study Design and Patients

In this single-center, non-randomized pilot study we investigated the feasibility of sentinel lymph node detection with ICG technique in patients with early OSCC. After institutional board review (Req-2022–00395) we enrolled five patients with histopathological proven oral cavity malignancy with a T1 or T2 and cN0 stage. Clinical stage was based on physical examination and imaging studies (CT or MRI, neck ultrasound with ultrasound-guided fine needle aspiration biopsy as needed). For all patients, both conventional ^99m^Tc based and ICG based SLN biopsies were performed. Exclusion criteria were pregnancy, allergy to iodine, known intolerance to ICG, previous neck surgery or radiotherapy or recurrent cancer. The study was conducted in accordance with the Declaration of Helsinki. Informed consent for further use of patient related data and material was obtained from all included patients.

### SLN Biopsy Technique with ^99m^Tc Radiolabelled Human Albumin Nanocolloid and ICG-Injection

**Fig. 1:**
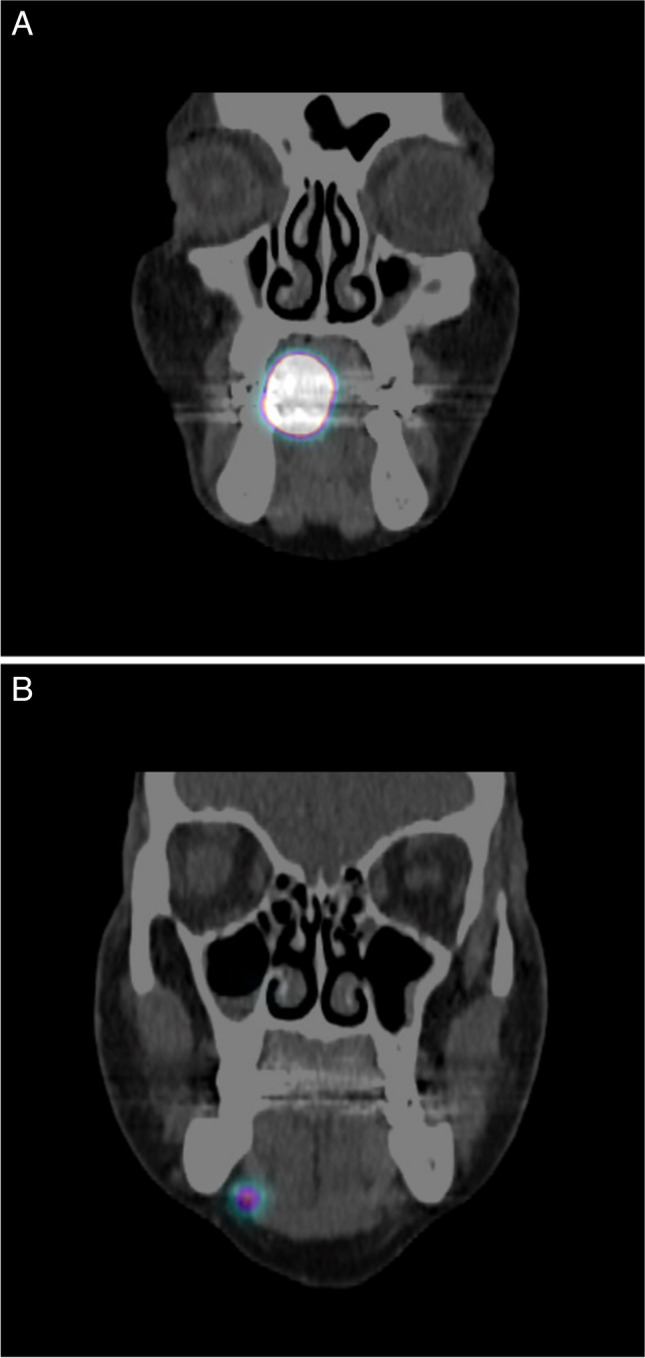
SPECT/CT of patient 5 with visible primary tumor (**A**) and one SLN (**B**) after injection of ^99m^Tc radiolabelled human albumin nanocolloid

Our head and neck multidisciplinary team (MDT) adheres to the National Comprehensive Cancer Network (NCCN) guidelines [23] for the treatment of our patients. After diagnosis and treatment recommendation by our head and neck MDT a resection of the primary tumor and SLN biopsy was performed in all patients [18]. The SLN biopsy technique with radioisotopes with injection of ^99m^Tc was performed preoperatively in the awake patient by the head and neck surgeon. We injected the ^99m^Tc radiolabelled human albumin nanocolloid (^99m^Tc NanoHSA-ROTOP (ROTOP Pharmaka AG, Dresden, Germany)) at four places into the adjacent mucosa around the primary tumor. A range between 70 and 113 MBq per patient was injected. Immediately after the radiotracer injection lymphoscintigraphy is started to show the drainage pattern and a SPECT/CT is performed by the nuclear medicine specialist. All areas of focal increased uptake outside the primary injection site were considered as SLN (Fig. 1). Then, surgery was carried out about 2–4 h after the SPECT/CT.

**Fig. 2:**
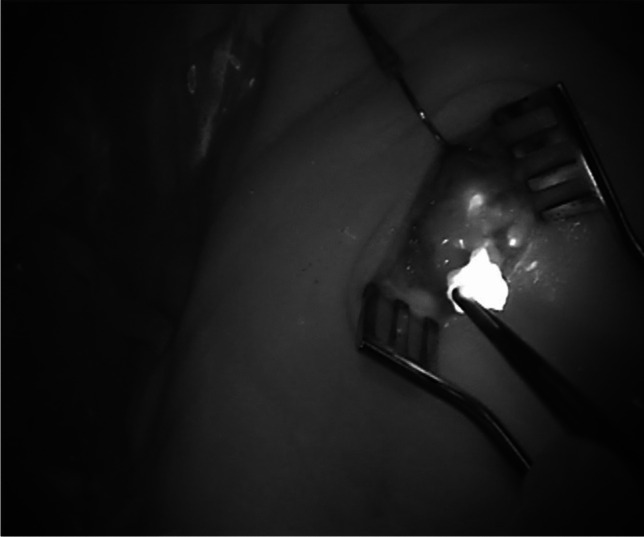
Intraoperative indocyanine green near-infrared imaging in patient 5 after raising the skin-platysma-flaps

In the operating theatre the ICG-dye (Verdye ®, Diagnostic Green GmbH, Germany) was injected around the primary tumor after anesthesia induction with the patient asleep into the same locations as the ^99m^Tc NanoHSA has been injected preoperatively. The ICG-dye is supplied as a water soluble, sterile and powder and was prepared before application with aqua ad iniectabila (2.5 mg/mL, 25 mg of ICG was diluted into 10 ml of aqua ad injectabila). An amount of 1–2 mL of this solution was injected around the tumor. Subsequently excision of the primary tumor and the neck incision was performed according to the preoperative SPECT/CT images which were available in the operating theatre to expose the SLN prior detected on SPECT/CT. The standard subplatysmal flaps were raised. With the thus exposed neck, the area of the sentinel lymph node(s) was detected with the hand-held gamma probe, the sentinel lymph nodes were then made visible with the ICG and near-infrared fluorescence imaging (Fig. 2). As gamma probe we used Neoprobe Gamma Detection System, Model 2300 (Neoprobe Corporation, OH). Time between injection of the ICG and intraoperative visualization of the SLN was between 30 and 60 min. We used the Fluobeam-System (Fluoptics, Grenoble, France) to perform the real-time near-infrared fluorescence imaging and detection of the ICG-marked lymph nodes. To improve the contrast and detection of the lymph nodes, the operating room lights were temporarily dimmed. We only categorized the lymph nodes into fluorescent or non-fluorescent and did not conduct quantification of the fluorescence of the lymph nodes. All detected SLNs—the ones found to have radioactivity and fluorescence as well as the ones found to have fluorescence only—were excised and sent to histopathological examination according to the sentinel lymph node dissection protocol. The SLN were considered as cleared once the surgical bed was free of radiation and ICG-containing nodes, as per screening with the hand-held gamma probe and near-infrared fluorescence imaging.

## Results

**Fig. 3:**
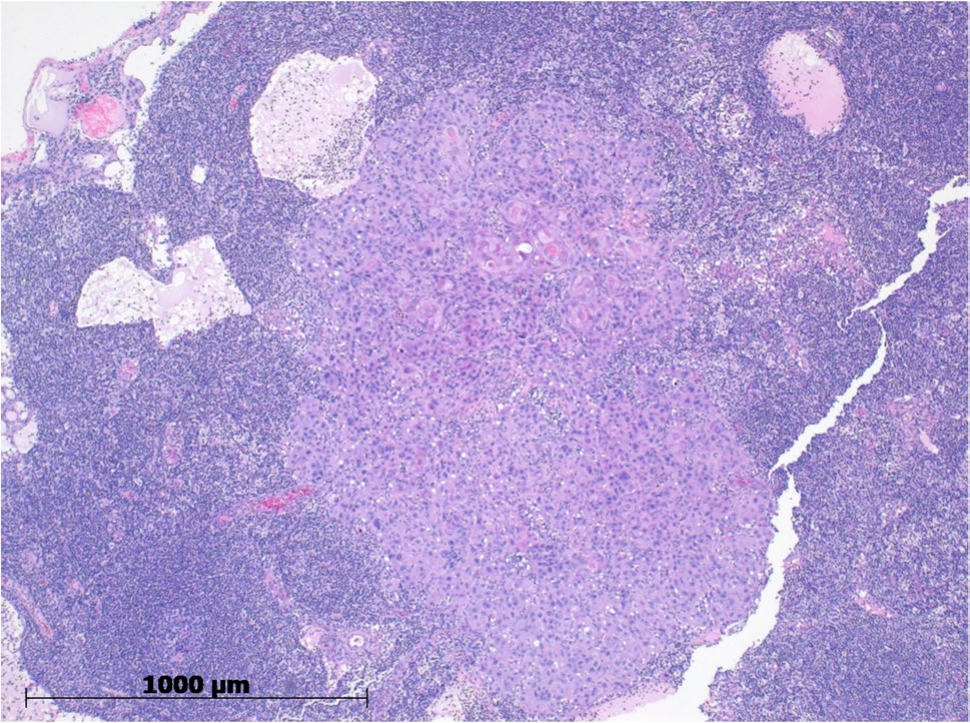
Histopathological image of the metastasis in the SLN of patient 5

We included five patients with early OSCC in this pilot study between June 2021 and August 2022. Three of the five patients were male and mean age at surgery was 61.6 years (SD 10.85). Preoperative T stage was cT1 in 3 patients, and cT2 in 2 patients. All the patients had a clinically node-negative lymph node status and no evidence of distant metastasis preoperatively. In all patients we conducted a radical resection of the primary tumor with negative surgical margins after injection of ^99m^Tc and ICG. In all patients primary closure was possible with no need for surgical reconstruction. In three of the five patients, the T category had to be upstaged postoperatively due to depth of invasion or size of greatest dimension. In two patients occult lymph node metastasis was found in one SLN. Both histopathological positive SLN were detected with ICG and the radioisotope method. Figure 3 shows the histopathological image of the metastasis found in the SLN of Patient 5. Further patients baseline characteristics are described in Table 1.

**Table 1:** Baseline characteristics of all 5 included patients with tumor site and TNM-stage

Patient	Age	Sex	Tumor site	cTNM	pTNM
1	59	male	Oral tongue	cT1 cN0	pT2 pN0
2	68	male	Floor of mouth	cT2 cN0	pT3 pN0
3	77	female	Oral tongue	cT1 cN0	pT1 pN0
4	53	male	Oral tongue	cT2 cN0	pT3 pN1 (1/18, 1/2 sn)
5	51	female	Oral tongue	cT1 cN0	pT2 pN1 (4/42, 1/1 sn)

The SLN could not be visualized transcutaneous with near-infrared fluorescence imaging in all five patients. After performing the standard subplatysmal flaps according to the preoperative SPECT/CT the SLN were made visible with ICG and were detected with the hand held gamma probe. Table 2 shows the number of sentinel lymph nodes detected by the two techniques used. At least one SLN was detected in all patients. With the radioisotope technique, we identified 7 sentinel lymph nodes. Intraoperatively all SLN identified with the radioisotope technique were also fluorescent and detected with ICG technique. The ICG injection around the periphery of the primary tumor intraoperatively and near-infrared fluorescence imaging after skin incision and initial tissue preparation of the neck, allowed the identification of two additional fluorescent lymph nodes, resulting in a total of 9 excised lymph nodes. In both of the two additional detected lymph nodes no occult metastasis was found. A mean of 1.8 (SD 0.45) lymph nodes was found in each patient. All the SLN were detected in the ipsilateral neck. Intraoperatively we performed a frozen section in two patients. In patient 2 the frozen section and definitive histopathology did not show any occult metastasis. In patient 4 the lymph node metastasis was diagnosed on frozen section and a unilateral neck dissection of the levels I to III was performed during the same anesthesia. In patient 5 a staged completion unilateral neck dissection of the levels I to IV was performed after detection of a metastasis in the sentinel lymph node on final histopathology. There were no adverse effects from the use of the ICG-dye intraoperatively. There were also no surgical complications noted during our pilot study.

**Table 2:** The number of detected (sentinel) lymph nodes by radioisotope technique and ICG-method per patient and total number of the detected lymph nodes

Patient	Total of SLNs	SLNs identified by radioisotope technique (*)	SLNs identified by ICG	Positive SLN on histopathological examination
1	2	2	2	0
2	2	1	2	0
3	2	2	2	0
4	2	1	2	1
5	1	1	1	1
Total	9	7	9	2

(*) All SLN identified by radioisotope techniqe were fluorescent as well

During the follow-up time three of the five included patients were tumor-free, one died due-to another malignancy and one patient showed a local recurrence on the tongue.

## Discussion

This was a single-center pilot study with five OSCC patients undergoing SLN biopsy with conventional radioisotope technique and ICG-technique, which showed that SLN biopsy with ICG near-infrared imaging is basically a feasible and reliable method to detect the SLN in early OSCC patients.

In our study with the ICG-technique we found two lymph nodes in addition to the 7 SLN detected by the conventional radioisotope technique, this concerts with to the results by Nakamura et al. [19]. Christensen et al. [24] showed that the majority of the additional detected lymph nodes with ICG were located in level I, close to the primary tumor. The detection of SLN with use of the gamma-probe can be difficult due to the shine-trough-effect especially in floor of mouth lesions. The average number of SLN per patient was 1.8, which was a bit lower than in previous studies who detected a mean of 2—3.4 SLN with ICG [19, 25, 26]. Our results and the current literature suggest that the additional usage of ICG and near-infrared imaging in combination with radiotracers simplifies and increases the intraoperative detection of the SLN through better visualization and resolution via ICG and this therefore allows to identify more lymph nodes than with the radioisotope method alone [19, 24, 27]. Additionally Nakamura et al. showed a tendency of shorter operating time when adding the ICG tracer for intraoperative detection [19]. This applies especially where shine-trough-effects can lead to difficulty of detection of the SLN with the use of a gamma-probe [24, 27].

In two of the five patients we found occult lymph node metastasis in one of the excised SLN, where in the other three patients SNL biopsies were negative on histopathology. This corresponds to the knowledge that that in up to 30% of early OSCC occult metastasis can be found and that SLN biopsies can detect these occult metastasis [2, 28].

ICG is a small molecule and binds intravascularly to plasma proteins, mostly albumin and alpha- and beta-lipoproteins and is transported quickly to the SLN, this allows for the quick visualization of the SLN within a few minutes after injection, and the ICG-injection to be done after anesthesia induction with the patient asleep thus avoiding the pain morbidity related to the injection in the awake patient [19]. The molecule size, however, is an important factor for the distribution with smaller molecules being transported and distributed faster potentially also into higher echelon nodes. This may lead to a overestimation of the number of (sentinel) lymph nodes detected with ICG [19], corresponding to our finding of two additional detected lymph nodes with near-infrared fluorescence imaging, compared with the radioisotope method. Especially with longer time between injection and detection of the lymph nodes [21]. Another factor for optimal visibility is the concentration and volume of the injected ICG. A Meta-analysis from 2014 suggested improved lymphatic drainage imaging with lower concentration and larger volume to the injected ICG-solution [29]. Furthermore Markuszewski et al. showed that the quality of visualization correlates with the ratio of albumin and ICG. If there is an excess of ICG, free dye is present what results in imprecise images of the SLN. And that higher and longer luminescence could be achieved with use of ICG bound with a human serum albumin [30]. Another possibility is to use hybrid tracer (ICG-^99m^Tc-nanocolloid) to avoid the transport of the ICG-tracer to higher echelon lymph nodes or leakage into the surgical field [31]. It is known that an ideal tracer should combine rapid transport and durable retention in the SLN, and that the distribution of the injected particles depends on the particle size and stability of the administered colloid particles as it was studied for radionuclides in the context of SLN biopsy. Therefore, no valid SLN biopsy can be performed by using small particles (smaller than 4 – 5 nm), as they penetrate the capillary membranes and therefore are not migrating trough the lymphatic channels and are dispersed into the vascular system. Neither with usage of large particles (500 – 2000 nm), as they stay trapped at the injection site and do not enter the lymphatic system [32, 33]. We used ^99m^Tc NanoHSA, i.e. radionuclide bound to human serum albumin with a particle size that drain fast into the lymph nodes and accumulate there within 10 min and are large enough to stay in the first draining nodes and not to overflow in higher echelon lymph nodes [2]. In more than 95% the particles of the nanocolloidal albumin are smaller than 80 nm, around 4% have a size of 80 – 100 nm and only 1% is larger than 100 nm [32, 33]. Further studies for better understanding in the transportation and distribution of the ICG-molecules and for determination of the best concentration and amount of injected ICG-solution are needed.

ICG as a new tracer and near-infrared imaging for detection of SLN has gained importance in recent years. ICG is free of radiation and has almost no side effects and risks. We were also able to confirm this in our study, as no side effects occurred after peritumoral injection of the ICG. There is no need for preoperative injection as this method is a one-step procedure with injection of the primary lesion during anesthesia which makes this technique of SLN biopsy more convenient and far less morbid for the patient. Other advantages of SLN biopsy with ICG are the lower costs of the tracer compared with radiotracers, no need for a nuclear physician and no exposure of the patient and medical personnel to radiation [25]. In addition, the shine-through-effect which typically occurs in radioisotope SLN mapping for floor of mouth cancers does not exist with the ICG-SLN biopsy [19, 25].

With the possibility of the intraoperative injection of the ICG under general anesthesia, the use of the ICG and therefore SLN biopsy could potentially be extended to other tumor sites of the head and neck such as laryngeal, hypopharyngeal or thyroid malignancies which are currently not accessible for preoperative injection with radioisotopes and subsequent SLN biopsy.

One of the limitations of our study was and of the usage of ICG for SLN biopsy in general is, that we were not able to visualize the SLN by ICG and near-infrared imaging through the skin without a neck incision. It is known that current disadvantages of the ICG imaging are its limited depth of penetration of 0.5 – 1.5 cm through the skin and subcutaneous tissue [34, 35]. This means that the lymph nodes cannot always be visualized transcutaneously without previous neck incision. Especially in OSCC, where the lymphatic channels and the first draining lymph nodes are usually located in the deep fatty tissue of the neck and are often blocked by the mandible or the sternocleidomastoid muscle. This has also been shown in a study from 2012, where only 12% of the SLN were detectable with only near-infrared imaging and the majority of these detected SLN with ICG only were located in Level I, where the shine through effect affects detectability of the sentinel lymph nodes with the radioisotope method [24], However, Nakamura et al. showed good transcutaneous visualization of the sentinel lymph nodes by applying pressure on the skin [19]. One possible factor for better visualization transcutaneously could be the BMI of the individual patient as Christensen et al. showed an association with lower BMI successful visualization transcutaneously [24].

As a result of these disadvantages of the ICG-method, the above-mentioned advantages of the ICG (no pain for the patient during injection, no need for a nuclear specialist, no radiation exposure, lesser costs) are not yet as significant, as for the moment the ICG can only be used in combination with the radioisotope method for SLN biopsy in OSCC.

Another limitation of our study is the small number of patients included as this was a pilot study to investigate the feasibility of the ICG-technique for SLN in head and neck tumors.

In future, ICG-guided sentinel lymph node biopsy could be used in a complementary fashion for faster intraoperative detection and better visualization of lymph nodes in addition to the current SLN biopsy with preoperative injection of radioisotopes and SPECT/CT. With technical progress and better transcutaneous visualization the ICG-guided sentinel lymph node biopsy could possible replace the conventional SLN biopsy in the future, but this needs to be validated with prospective studies which are currently underway at our unit.

## Conclusion

We demonstrated that SLN biopsy with ICG near-infrared imaging is a feasible and valid method for detection of sentinel lymph nodes, especially in combination with current state of the art method with preoperative injection of radioisotope and SPECT/CT. The ICG technique provides a better and faster visualization and thus easier intraoperative detection of the SLN. The ICG-SLN technique could be extended to other regions of the head and neck which are currently not accessible through injection in the awake patient.

## Data Availability

The datasets generated for this study can be obtained upon reasonable request by email to the corresponding author.
